# Specific Nested PCR for the Detection of 16SrI and 16SrII Group Phytoplasmas Associated with Yellow Leaf Disease of Areca Palm in Hainan, China

**DOI:** 10.3390/plants14142144

**Published:** 2025-07-11

**Authors:** Huiyuan Ge, Xiuli Meng, Zhaowei Lin, Saad Jan, Weiwei Song, Weiquan Qin, Qinghua Tang, Xiaoqiong Zhu

**Affiliations:** 1 Department of Plant Pathology and MARA Key Laboratory of Pest Monitoring and Green Management, College of Plant Protection, China Agricultural University, Beijing 100193, China; gehuiyuan29@gmail.com; 2 Coconut Research Institute/HPDST Hainan Innovation Center of Academician Team, Chinese Academy of Tropical Agricultural Sciences, Wenchang 571339, China; mengxl2020@126.com (X.M.); linzhaowei163@163.com (Z.L.); drsaadjan@bkuc.edu.pk (S.J.); songweiwei426@aliyun.com (W.S.); qwq268@163.com (W.Q.); 3 Sanya Institute of China Agricultural University, Sanya 572000, China

**Keywords:** *Areca catechu* L., areca palm yellow leaf phytoplasma, molecular detection

## Abstract

Yellow leaf disease (YLD), caused by the areca palm yellow leaf phytoplasma (APYL), poses a significant threat to the sustainability of the areca palm industry. Timely and accurate detection is essential for effectively diagnosing and managing this disease. This study developed a novel nested PCR system using primers specifically designed from conserved regions of the phytoplasma 16S rDNA sequence to overcome limitations such as false positives often associated with universal nested PCR primers. The resulting primer pairs HNP-1F/HNP-1R (outer) and HNP-2F/HNP-2R (inner) consistently amplified a distinct 429 bp fragment from APYL strains belonging to the 16SrI and 16SrII groups. The detection sensitivity reached 7.5 × 10^−7^ ng/μL for 16SrI and 4 × 10^−7^ ng/μL for 16SrII. Field validation using leaf samples from symptomatic areca palms confirmed the high specificity and reliability of the new primers in detecting APYL. Compared to conventional universal primers (P1/P7 and R16mF2/R16mR1), this newly developed nested PCR system demonstrated higher specificity, sensitivity, and speed, making it a valuable tool for the early diagnosis and management of YLD in areca palms.

## 1. Introduction

Areca palm (*Areca catechu* L.) is a perennial evergreen species belonging to the genus Areca within the family Palmaceae. Originally native to Malaysia, it is now widely cultivated throughout South and Southeast Asia, spanning 16 countries and regions, including India and Malaysia [1]. Its primary cultivation areas in China are Hainan and Taiwan Provinces, with smaller-scale production in Yunnan, Guangdong, and Guangxi Provinces [2]. The areca palm is valued for its dietary and medicinal properties, including applications in treating various human ailments [3]. It is recognized as one of Hainan’s key crops, part of the province’s so-called “Six Trees”, and accounts for over 95% of the total area of areca cultivation in China [4]. Currently, the areca palm industry supports the livelihoods of more than 2.3 million farmers in Hainan [5], contributing significantly to regional agricultural development, rural revitalization, and income generation for farming communities.

Yellow leaf disease (YLD) of areca palm, caused by the areca palm yellow leaf phytoplasma (APYL), is a highly destructive condition first identified in 1914 in central Kerala, India [6]. Subsequent occurrences were reported in China in 1981 [7] and in Sri Lanka in 2015 [8]. In China, the disease was initially detected in Tunchang County, Hainan Province, and has since spread to nearly all areca-growing regions across the island, inflicting significant losses on the local areca palm industry [5,9]. Without effective control strategies in cultivation practices, early and accurate pathogen detection remains critical for effective disease management.

In India, three distinct ‘*Candidatus* Phytoplasma’ species have been identified in association with YLD: ‘*Ca*. P. asteris’ (16SrI-B), ‘*Ca*. P. sacchari’ (16SrXI-B), and ‘*Ca*. P. cynodontis’ (16SrXIV-A) [10,11,12]. However, previous studies in China reported that YLD-associated phytoplasmas primarily belonged to the 16SrI-B and 16SrI-G subgroups [13,14]. More recently, other phytoplasma groups 16SrII and 16SrXXXII have been detected in YLD-affected areca palm samples collected from Hainan [15,16].

To date, a range of detection methods has been developed for identifying APYL, including electron microscopy [17], enzyme-linked immunosorbent assay (ELISA) [18], nested PCR [14,19], loop-mediated isothermal amplification (LAMP) [20], droplet digital PCR (ddPCR) [21], and quantitative PCR (qPCR) [22,23]. While each technique offers specific advantages, they also present significant limitations. For example, electron microscopy is labor-intensive, requires complex sample preparation, and incurs high costs. Serological methods such as ELISA may suffer from challenges in antisera production and risks of cross-reactivity with host antigens. Although LAMP enables isothermal amplification, its complicated primer design [24] and susceptibility to contamination can result in false positives. In comparison, qPCR and ddPCR provide high sensitivity but demand costly reagents, sophisticated instrumentation, and stricter laboratory conditions. Moreover, products generated by ELISA, LAMP, or qPCR are not suitable for sequencing.

Nested PCR using universal phytoplasma primers remains a widely adopted method for APYL detection and classification, primarily because the amplified 16S rDNA fragments can be sequenced. However, this approach has drawbacks, particularly the risk of false-positive results due to non-specific amplification, which can significantly compromise detection accuracy [25]. To address issues related to the low concentration and uneven distribution of APYL in host tissues, a nested PCR primer set (F4/R1 and F2/R2 [20]) was previously developed, showing improved sensitivity over traditional universal primer sets such as P1/P7 and R16mF2/R16mR1 or P1/P7 and R16mR2n/R2 [4,26]. However, studies have shown that this primer set could generate non-specific amplification products.

Recent surveillance in Hainan Province revealed that phytoplasmas of the 16SrI group are prevalent, with increasing reports of 16SrII group detection in regions such as Wenchang and Danzhou. The 16SrXXXII group has been observed in only a single case [16]. Currently, no existing diagnostic method can simultaneously detect both 16SrI and 16SrII APYL strains.

This study developed a novel nested PCR primer pair combination to overcome the challenge of non-specific amplification and enable concurrent detection of both 16SrI and the newly identified 16SrII APYL groups. This new system offers high specificity and sensitivity for APYL detection across both phytoplasma groups in Hainan, thus providing a reliable diagnostic tool to support early identification and control of yellow leaf disease in areca palm seedlings and field-grown plants.

## 2. Results

### 2.1. Application of Universal Nested PCR for Phytoplasma Detection

**Figure 1:**
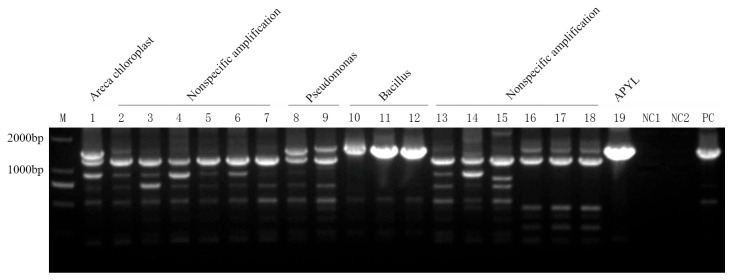
Profile of 16S rDNA sequences amplified by nested PCR with universal primer P1/P7 followed by R16mF2/R16mR1, from areca samples. M: DL2000 DNA Marker; 1–19: areca samples; NC1: blank control; NC2: blank control of nested PCR; PC: ‘*Candidatus* Phytoplasma asteris’-related strain H-9.

**Figure 2:**
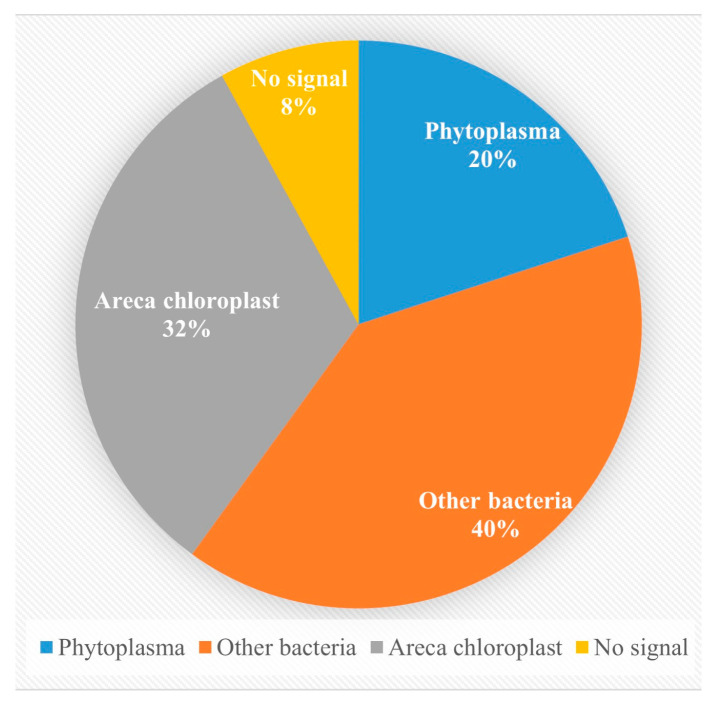
Ratios of different sequences in 50 samples.

A total of 335 genomic DNA samples from areca palm were screened using the universal nested PCR primer set P1/P7, followed by R16mF2/R16mR1, targeting phytoplasma 16S rDNA (Table A1). Among these, 50 samples produced amplification bands of approximately 1400 bp (Figure 1). The nested PCR products from these 50 putative positives were then sequenced by Sangon Biotech (Shanghai, China). Sequence analysis revealed the following: 16 samples corresponded to areca palm chloroplast DNA, 20 matched the bacterial sequences, only 10 were confirmed as being phytoplasma-specific (Table A1 NO. 1–10), and 4 samples failed to yield any usable sequencing data (Figure 2).

### 2.2. Nested PCR Primer Test and Combination

**Figure 3:**
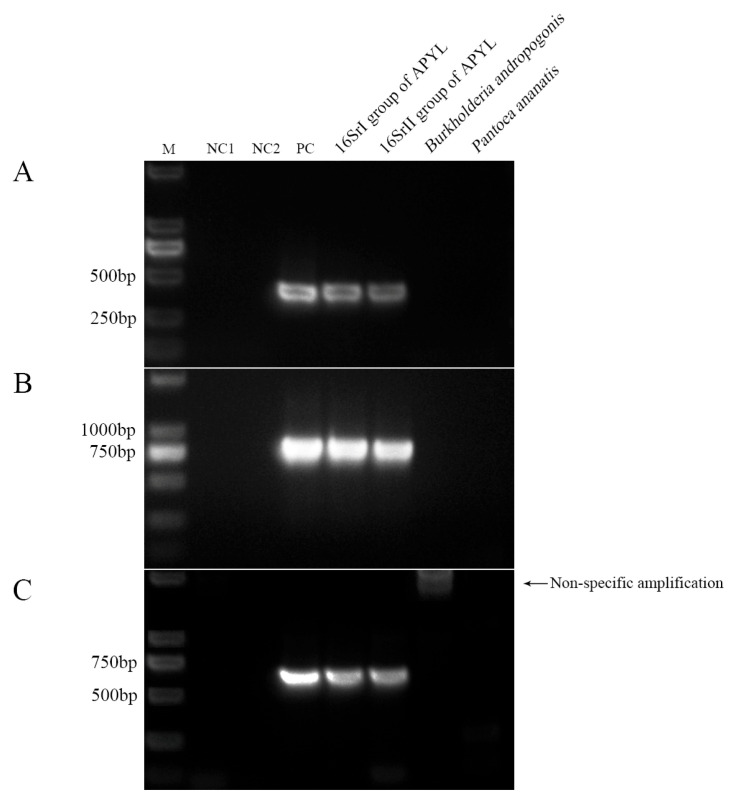
This study conducted a preliminary screening of three pairs of internal primers. (**A**) 16S rDNA sequences amplified by nested PCR with primer HNP-1F/1R followed by HNP-2F/2R; (**B**) 16S rDNA sequences amplified by nested PCR with primer HNP-1F/1R followed by HNP-3F/3R; (**C**) 16S rDNA sequences amplified by nested PCR with primer HNP-1F/1R followed by HNP-4F/4R. M: marker DL2000; NC1: blank control; NC2: blank control for nested PCR; PC: ‘*Candidatus* Phytoplasma asteris’-related strain H-9.

Based on multiple sequence alignments of 16S rDNA from APYL, areca chloroplasts, and pathogenic and endophytic bacteria, and guided by established PCR primer design principles, one outer primer pair (HNP-1F/HNP-1R) and three internal primer pairs (HNP-2F/2R, HNP-3F/3R, and HNP-4F/4R) were designed (Table 1). These nested PCR primers were evaluated using genomic DNA from areca palm leaves infected with 16SrI and 16SrII phytoplasma groups and DNA from *Burkholderia andropogonis* and *Pantoea ananatis* as reference strains. Among the internal primer sets, HNP-2F/2R and HNP-3F/3R specifically amplified target bands only in the positive control and areca palm samples infected with 16SrI or 16SrII phytoplasmas (Figure 3A,B). However, HNP-4F/4R also produced a ~1500 bp band from the DNA of the bacterial leaf blight pathogen (Figure 3C), indicating non-specific amplification. Therefore, for subsequent nested PCR assays, HNP-1F/1R was paired with either HNP-2F/2R or HNP-3F/3R.

### 2.3. Specificity Validation of Nested PCR

**Figure 4:**
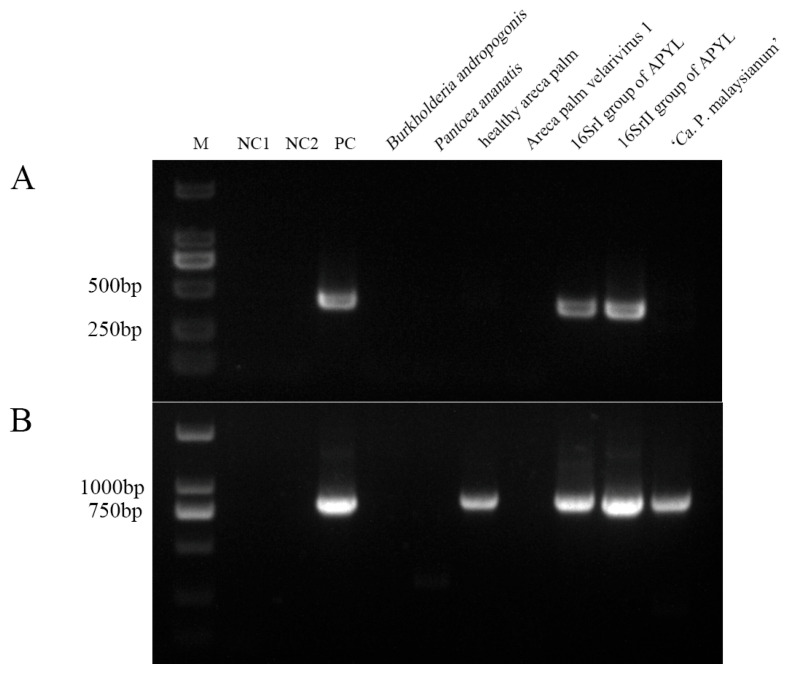
Profile of 16S rDNA sequences amplified by nested PCR with primer HNP-1F/1R followed by HNP-2F/2R and HNP-3F/3R, respectively, for the specificity validation. (**A**) HNP-2F/2R, (**B**) HNP-3F/3R. M: marker DL2000; NC1: blank control; NC2: blank control for nested PCR; PC: ‘*Candidatus* Phytoplasma asteris’-related strain H-9; Lane 5: *Burkholderia andropogonis* (*Robbsia andropogonis*); Lane 6: *Pantoea ananatis*; Lane 7: healthy areca leaf sample; Lane 8: Areca palm velarivirus 1; Lane 9: APYL of 16SrI group; Lane 10: APYL of 16SrII group; Lane 11: ‘*Candidatus* Phytoplasma malaysianum’ strain SHM-1.

To evaluate detection specificity, the outer primers HNP-1F/1R were paired with internal primers HNP-2F/2R and HNP-3F/3R and tested against genomic DNA from areca palm samples infected with 16SrI and 16SrII phytoplasmas, as well as DNA from five other pathogens. Results showed that the HNP-2F/2R primer pair consistently amplified a specific band of approximately 429 bp only in the positive control and in areca palm samples infected with 16SrI and 16SrII phytoplasmas (Figure 4A). However, the HNP-3F/3R set also amplified a 652 bp fragment in healthy areca palm samples and those infected with 16SrXXXII group phytoplasmas (Figure 4B), indicating reduced specificity. Therefore, HNP-2F/2R was selected as the internal primer pair for all subsequent assays.

### 2.4. Optimization of Nested PCR Annealing Temperatures

**Figure 5:**
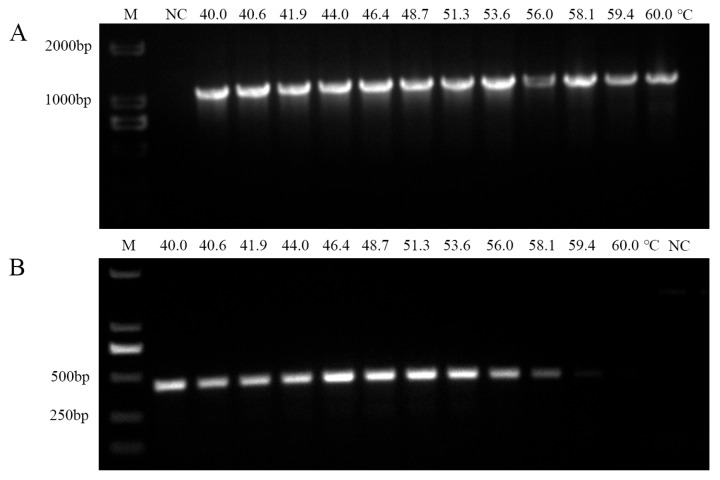
The HNP-1F/1R + HNP-2F/2R primer set was used, targeting the ‘*Candidatus* Phytoplasma asteris’-related strain H-9. (**A**) Annealing temperature optimized for the outer primer HNP-1F/1R. M: marker DL2000; NC: blank control. M: marker DL2000; NC: blank control. (**B**) Annealing temperature optimized for the inner primer HNP-2F/2R. M: marker DL2000; NC: blank control.

In the nested PCR assays, annealing temperatures for both outer and internal primer sets were systematically optimized across a gradient from 40 °C to 60 °C. For the outer primer pair HNP-1F/1R, no significant differences in amplification intensity were observed between 40.0 °C and 53.6 °C. However, as the temperature increased from 56.0 °C to 60.0 °C, a marked decline in band intensity was noted (Figure 5A), leading to the selection of 53.6 °C as the optimal annealing temperature.

For the internal primers HNP-2F/2R, a temperature gradient analysis revealed consistent amplification of the target band within the range of 46.4 °C to 51.3 °C, while no detectable amplification occurred at 60.0 °C (Figure 5B). Based on these observations, 51.3 °C was chosen as the optimal annealing temperature for the HNP-2F/2R primer set.

### 2.5. Sensitivity Determination of Nested PCR

To evaluate the sensitivity of the developed method for detecting phytoplasmas from the 16SrI and 16SrII groups, nested PCR was performed using the outer primers HNP-1F/1R and internal primers HNP-2F/2R on recombinant plasmids harboring 16S rDNA fragments specific to each group.

**Figure 6:**
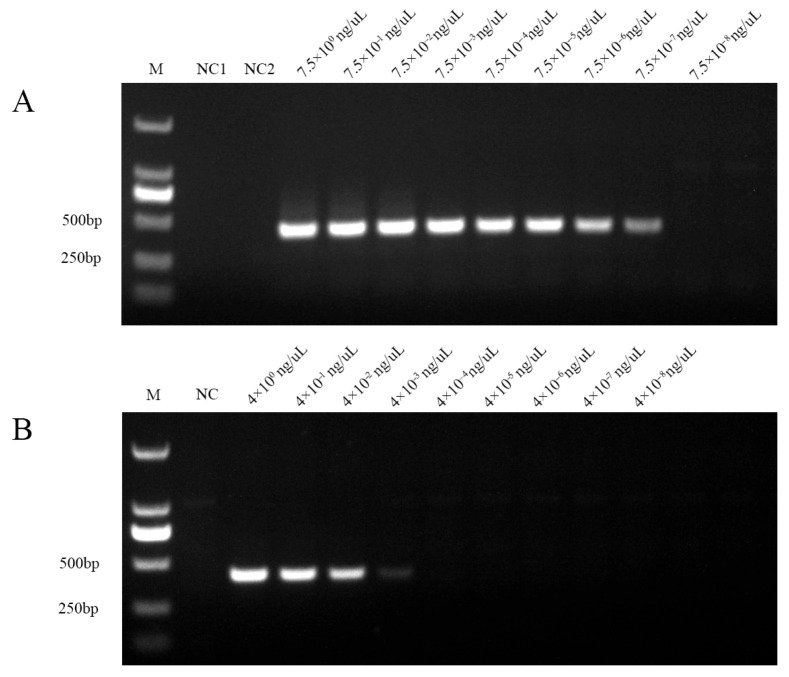
Sensitivity test for nested PCR primer set of HNP-1F/1R and HNP-2F/2R. (**A**) Gel electrophoresis for the sensitivity of 16SrI phytoplasma using a developed nested PCR. M: marker DL2000; NC1: blank control; NC2: blank control for nested PCR. (**B**) Gel electrophoresis results for the sensitivity of 16SrII phytoplasma using developed nested PCR. M: marker DL2000; NC: blank control.

For the 16SrI group, a distinct and specific amplification band was consistently observed across a plasmid concentration range from 7.5 ng/μL down to 7.5 × 10^−7^ ng/μL. As the concentration decreased, the intensity of the amplification band gradually weakened and became undetectable at 7.5 × 10^−7^ ng/μL (Figure 6A). Similarly, in the case of the 16SrII group, a clear target band of 429 bp was stably amplified within the range of 4 ng/μL to 4 × 10^−3^ ng/μL, with diminishing intensity as the plasmid concentration decreased and no amplification detected at 4 × 10^−3^ ng/μL (Figure 6B).

These findings demonstrate that the nested PCR assay has a detection limit of 7.5 × 10^−7^ ng/μL for the 16SrI group and 4 × 10^−7^ ng/μL for the 16SrII group of phytoplasmas.

### 2.6. Comparison of the Developed Nested PCR to the Universal Nested PCR

To evaluate the detection efficiency of the newly developed nested PCR method compared to the conventional universal nested PCR approach, 30 areca palm samples collected from the field were tested (Table 2).

**Figure 7:**
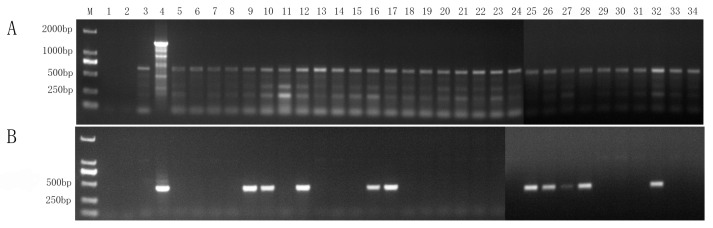
Comparison of bands amplified using universal primers (P1/P7 and R16mF2/R16mR1) with the primers developed in this study. (**A**) universal primers; (**B**) the primers developed in this study; M: marker DL2000; 1: blank control; 2: blank control for nested PCR; 3: healthy areca leaf sample; 4: ‘*Candidatus* Phytoplasma asteris’-related strain H-9; 5–34: areca leaf samples collected in the fields.

The universal nested PCR method using primer sets P1/P7 and R16mF2/R16mR1 detected phytoplasma only in the positive control sample and failed to identify any positive cases among the field samples (Figure 7A). However, the newly developed nested PCR method detected phytoplasmas in 10 of the 30 field samples (Figure 7B). The sequencing analysis of these 10 positive samples confirmed that all amplicons corresponded to phytoplasmas belonging to the 16SrI group. These results underscore the higher sensitivity and reliability of the specific nested PCR method established in this study for detecting APYL phytoplasmas, offering a valuable tool for routine surveillance and early diagnosis in areca palm plantations affected by yellow leaf disease.

### 2.7. Construction and Analysis of Phylogenetic Trees

**Figure 8:**
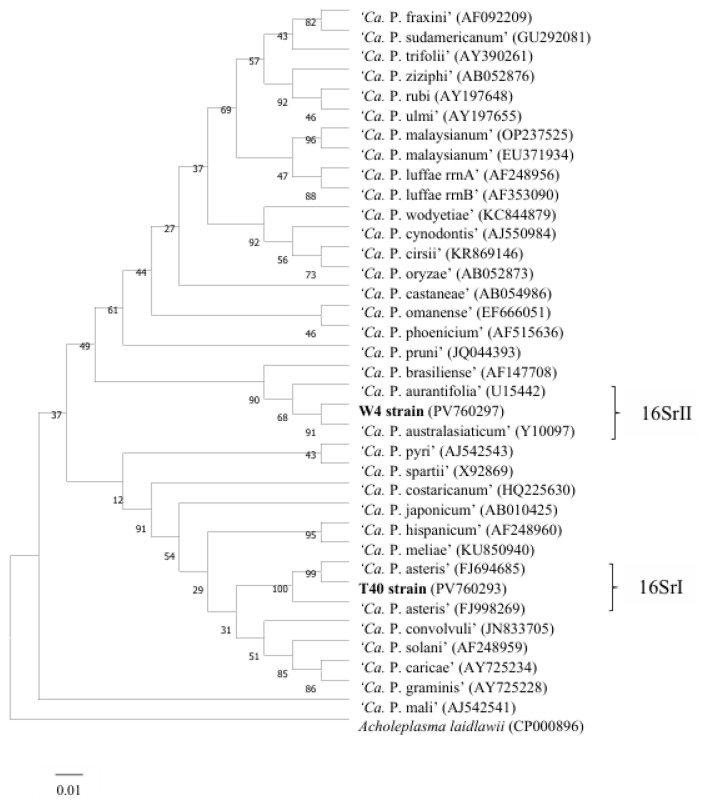
This is a phylogenetic tree constructed by the neighbor-joining method based on partial fragments of the phytoplasma 16S rRNA gene amplified by HNP-2F/2R. The scale bar length represents the inferred changes in character states. Branch lengths are proportional to the inferred number of character state transitions. The percentage of replicate trees in which the associated taxa clustered together in the bootstrap test (1000 replicates) is shown next to the branches.

Phylogenetic analysis was conducted based on the fragments amplified using the HNP-2F/2R primers. The obtained sequences were compared with 16S rRNA sequences of known phytoplasmas in the GenBank database. A phylogenetic tree was constructed using the neighbor-joining method in MEGA 11, with *Acholeplasma laidlawii* serving as the out-group. In the resulting tree, phytoplasmas from the 16SrI and 16SrII groups formed distinct clades. This demonstrates that the nested PCR method developed in this study can effectively distinguish and group phytoplasmas associated with yellow leaf disease in areca palm within the 16SrI and 16SrII groups (Figure 8).

## 3. Discussion

Phytoplasmas, previously referred to as mycoplasma-like organisms (MLOs) [30], are prokaryotes that lack a cell wall [31,32] and remain notoriously difficult to culture in artificial media [33]. In this study, we designed outer and internal primers targeting conserved 16S rDNA regions specific to the APYL phytoplasma. Among these, the primer set HNP-1F/HNP-1R and HNP-2F/HNP-2R was ultimately selected for its specificity in amplifying APYL. The annealing temperatures and nested PCR parameters were systematically optimized to ensure reliable detection.

Universal primers such as P1/P7 and R16mF2/R16mR1 are commonly employed for detecting phytoplasmas across diverse host species. However, in this work it was found that their non-specific amplification was quite notable. Our results indicated that the specificity of the universal primers P1/P7 and R16mF2/R1 for the amplification of APYL is relatively poor, resulting in a high false-positive rate (72% to 80%). This observation underscores their limited specificity in the context of the areca palm. A similar issue was noted in detecting phytoplasma associated with sisal purple leafroll disease [34]. In earlier field tests during 2018 and 2019, screening of 100 leaf samples with these universal primers yielded only one confirmed phytoplasma sequence, identified as ‘*Ca*. P. asteris’. A previous study introduced a nested PCR approach using primer sets F4/R1 and F2/R2 [20] to improve detection accuracy, targeting a 525 bp fragment. While this approach enhanced detection rates, subsequent analyses revealed non-specific amplification as a limitation.

The nested PCR method developed in the present study demonstrated high specificity, reliably detecting phytoplasmas from both the 16SrI and 16SrII groups currently identified in areca palms, without cross-reactivity to other phytoplasma groups or bacterial pathogens.

In terms of sensitivity, the method achieved a minimum detection threshold of 7.5 × 10^−7^ ng/μL for the ‘*Ca*. P. asteris’ strain (Figure 6A) and 4 × 10^−3^ ng/μL for the 16SrII phytoplasma group (Figure 6B). While qPCR and ddPCR offer lower detection limits, 1.16 copies/μL [23] and 0.07 copies/μL [22], respectively, our nested PCR approach presents a cost-effective alternative that does not compromise specificity or reliability. All amplified fragments obtained in this study were confirmed by sequencing to correspond to APYL phytoplasma.

In conclusion, the nested PCR method developed here provides a robust, economical, and specific tool for detecting and identifying APYL-associated phytoplasmas from the 16SrI and 16SrII groups. This approach offers valuable technical support for routine monitoring, early diagnosis, and effective areca palm yellow leaf disease management.

## 4. Materials and Methods

### 4.1. Materials

In this study, genomic DNA was extracted from field-collected areca palm leaf samples. After sequencing and confirmation of identity, the DNA samples were stored at −20 °C for use in primer specificity validation experiments (Table 3). Further leaf samples were also collected from the field, and their genomic DNA was similarly extracted and preserved at −20 °C. These samples were then used to compare the detection efficiency of the newly designed primers with that of conventional universal primers (Table 2).

### 4.2. Extraction of Total DNA from Areca Plam Leaf Samples Showing Yellow

Approximately 0.1 g of tissue was collected from the upper one-third of areca palm leaves, cut into 1 mm fragments, and ground using a cell disruptor. Genomic DNA was extracted using the Plant Genomic DNA Extraction Kit (TianGen Biotech, Beijing, China), quantified with a NanoDrop 2000 spectrophotometer, and stored at −20 °C for further use.

### 4.3. Construction of Recombinant Plasmids for 16S rDNA of Areca Palm Yellow Leaf Phytoplasma

In this study, universal nested PCR primers P1/P7 and R16mF2/R16mR1 (Table 1), targeting the 16S rDNA of phytoplasmas, were used to amplify total genomic DNA extracted from areca palm leaves showing YLD symptoms. Each 25 μL PCR reaction contained 2 μL of DNA template, 12.5 μL of 2× Taq PCR Master Mix (Aidlab Biotechnologies, Beijing, China), 1 μL of each primer (10 μM), and nuclease-free water to a final volume.

The PCR protocol involved an initial denaturation at 94 °C for 3 min, followed by 35 cycles of denaturation at 94 °C for 30 s, annealing at 55 °C for 30 s, extension at 72 °C for 1 min, and a final extension at 72 °C for 5 min. The PCR product (from P1/P7) was diluted 20-fold and used as the template for the nested PCR using internal primers R16mF2/R16mR1. This nested PCR followed the same cycling conditions, except for an annealing temperature of 50 °C.

For visualization, 5 μL of ethidium bromide (10 mg/mL; Sangon Biotech, Shanghai, China) was added to a 1% agarose gel prepared with 1× TAE buffer and mixed thoroughly by vortexing for 10 s. PCR products were electrophoresed at 120 V for 35 min and imaged using a gel documentation system (Synoptics, Cambridge, UK).

For recombinant plasmid construction, PCR products were purified using a gel extraction kit (Tiangen Biotech, Beijing, China), then ligated into a T-vector using a rapid ligation kit (Sangon Biotech, Shanghai, China). The ligation products were transformed into DH5α competent cells (Sangon Biotech, Shanghai, China). Positive colonies were screened in LB liquid medium containing 50 ng/μL ampicillin. Plasmid DNA was extracted using a plasmid mini-prep kit (Tiangen Biotech, Beijing, China) and sequenced by Sangon Biotech (Shanghai, China).

### 4.4. Design and Primary Screening of Specific Primers

In this study, the outer and inner primers were designed and screened to detect the 16S rDNA of phytoplasmas associated with YLD in areca palm. For primer design, previously reported 16S rDNA sequences from the 16SrI group (GenBank accession numbers: FJ998269 and FJ694685) and from the 16SrII group (GenBank accession number: OQ586085) were used. To improve specificity, these phytoplasma sequences were aligned and compared with those from Areca chloroplasts, known areca bacterial pathogens (e.g., *Burkholderia andropogonis* and *Pantoea ananatis*), and other bacterial species (Table 4). Based on sequence divergence, multiple nested PCR primers were designed and synthesized by Sangon Biotech (Shanghai, China).

For the initial amplification, genomic DNA extracted from areca palm leaves infected with 16SrI and 16SrII group phytoplasmas, as well as DNA from *Burkholderia andropogonis* and *Pantoea ananatis*, was subjected to PCR using the outer primer pair HNP-1F/1R. The ‘*Ca.* P. asteris’ strain (GenBank accession number: PV760299) was included as a positive control, and sterile water was a negative control. The resulting PCR products were diluted 20-fold and used as templates for nested PCR using three primer sets: HNP-2F/2R, HNP-3F/3R, and HNP-4F/4R.

### 4.5. Specificity Verification of Nested PCR

To assess the specificity of the newly developed method, the outer primer pair HNP-1F/1R was used to amplify genomic DNA from a range of sources, including healthy areca palm leaves, bacterial leaf spot pathogens of areca palm, pan-genus bacteria from pineapple, YLD-associated phytoplasmas from the 16SrI and 16SrII groups, and *Trema tomentosa* (Roxb.) Hara was infected with a 16SrXXXII group phytoplasma (GenBank accession number: PV759645). Complementary DNA from areca yellow leaf virus 1 (GenBank accession number: OQ423126) was also tested. The PCR products were diluted 20-fold and used as templates for a nested amplification using the internal primer sets described previously. The reaction system of nested PCR and cycling parameters followed those outlined in Section 4.3.

### 4.6. Optimization of Annealing Temperatures for Nested PCR with Internal Primers

To determine the optimal annealing temperature for nested PCR using internal primers for phytoplasma detection, the primer set HNP-1F/1R + HNP-2F/2R was employed, targeting the ‘*Candidatus* Phytoplasma asteris’ strain H-9 (GenBank accession number: PV760299).

For the PCR of amplification with primers HNP-1F/1R, the reaction mixture was identical to that described in Section 4.3. PCR cycling conditions consisted of an initial denaturation at 94 °C for 3 min, followed by 35 cycles of denaturation at 94 °C for 30 s, annealing at a temperature gradient from 40 °C to 60 °C in 12 increments (each for 30 s), extension at 72 °C for 1 min, and a final extension at 72 °C for 5 min.

Following this, the PCR products were diluted 20-fold and used as templates for nested amplification with primers HNP-2F/2R. The nested PCR reaction composition mirrored the PCR, with the same temperature gradient applied to optimize annealing conditions.

### 4.7. Sensitivity Testing of Developed Nested PCR

To evaluate the sensitivity of the nested PCR method developed in this study, recombinant plasmids S97 and W4, each harboring the 16S rDNA fragments from the 16SrI and 16SrII groups of APYL, respectively, were subjected to 10-fold serial dilutions. Plasmid S97 was diluted from 7.5 ng/μL to 7.5 × 10^−8^ ng/μL, and W4 from 4 ng/μL to 400 zg/μL. The nested PCR was performed using the HNP-1F/1R + HNP-2F/2R primer set to amplify the diluted plasmid templates. The reaction mixture and cycling conditions followed those outlined in Section 4.6.

### 4.8. Comparison of Developed Nested PCR Method with the Universal Nested PCR Method

In this study, 30 areca palm samples showing yellowing symptoms (Table 2) were simultaneously analyzed to compare the performance of two detection methods: one using universal primers and the other employing the newly developed nested PCR primer set. The universal nested PCR method was conducted according to the protocol detailed in Section 4.3, while the newly developed nested PCR assay followed the procedure described in Section 4.6.

### 4.9. Phylogenetic Analysis of the Fragment Amplified from Primers HNP1F/1R and HNP-2F/2R

The DNA fragments amplified by primers HNP-2F/2R were compared with 16S rRNA sequences of other phytoplasmas in the GenBank database. A phylogenetic tree was constructed using the neighbor-joining method in MEGA 11 software, with Acholeplasma laidlawii serving as the out-group and bootstrap analysis performed with 1000 replicates [34].

## Figures and Tables

**Table 1:** Primers for amplification of the 16S rDNA gene using nested PCR.

Primer	Primer Sequence (5′-3′)	Annealing Temperature (°C)	Target Fragment Length (bp)	References
P1	AAGAATTTGATCCTGGCTCAGGATT	55	1800	[27]
P7	CGTCCTTCATCGGCTCTT			[28]
R16mF2	CATGCAAGTCGAACGGA	50	1500	[29]
R16mR1	CTTAACCCCAATCATCGA			
HNP-1F	TTCTTGTTTTTAAAAGACCT	44	1072	This study
HNP-1R	AAACTTGCGCTTCAGCT			
HNP-2F	TGTGGTCTAAGTGCAAT	48	429	This study
HNP-2R	CTGATAACCTCCACTGTGTT			
HNP-3F	TTCTTGTTTTTAAAAGACCT	50	837	This study
HNP-3R	ATAACCTCCACTGTGTTTCT			
HNP-4F	AATGCTCAACATTGTGATGCT	48	652	This study
HNP-4R	AAACTTGCGCTTCAGCT			

**Table 2:** *Areca* leaf samples that were used for comparison between the two methods.

No	Sample	City/County	Year	No	Sample	City/County	Year
1	C002-1	Tunchang	2020	16	D093-2	Tunchang	2020
2	C007-2	Tunchang	2020	17	ZY2-1	Tunchang	2020
3	C013-2	Tunchang	2020	18	7-4-2	Tunchang	2020
4	C018Y-1	Tunchang	2020	19	003-2	Tunchang	2020
5	C018Y-2	Tunchang	2020	20	B021-2	Tunchang	2020
6	C021-1	Tunchang	2020	21	B027-2	Tunchang	2020
7	C023-1	Tunchang	2020	22	B029-2	Tunchang	2020
8	C026-1	Tunchang	2020	23	B030-1	Tunchang	2020
9	C026-2	Tunchang	2020	24	B032-1	Tunchang	2020
10	C031-2	Tunchang	2020	25	B037-2	Tunchang	2020
11	C042-1	Tunchang	2020	26	B038-2	Tunchang	2020
12	C064-1	Tunchang	2020	27	B042-1	Tunchang	2020
13	12-3-2	Tunchang	2020	28	B042-2	Tunchang	2020
14	D039-1	Tunchang	2020	29	B055-2	Tunchang	2020
15	D062-2	Tunchang	2020	30	B059-1	Tunchang	2020

**Table 3:** Plant leaf samples used in specificity verification of nested PCR.

Strain	Phytoplasma Identified	GenBank Accession No	Host Plant	Location
J069	‘*Ca*. P. asteris’ 16SrI	PV760294	Areca palm *Areca catechu*	Wenchang, Hainan
S97	‘*Ca*. P. asteris’ 16SrI	PV760296	Areca palm *A*. *catechu*	Wenchang, Hainan
T40	‘*Ca*. P. asteris’ 16SrI	PV760293	Areca palm *A*. *catechu*	Wenchang, Hainan
H-9	‘*Ca*. P. asteris’ 16SrI	PV760299	Periwinkle *Catharanthus roseus*	Wenchang, Hainan
A056	‘*Ca*. P. australasiae’ 16SrII	PV760298	Areca palm *A*. *catechu*	Wenchang, Hainan
W4	‘*Ca*. P. australasiaticum’ 16SrII	PV760297	Areca palm *A*. *catechu*	Wenchang, Hainan
SHM-1	‘*Ca*. P. malaysianum’ 16SrXXXII	PV759645	*Trema tomentosa*	Qionghai, Hainan
MW1-1	*Burkholderia andropogonis*	PV759631	Areca palm *A*. *catechu*	Wenchang, Hainan
I027	Areca palm velarivirus 1	OQ423126	Areca palm *A*. *catechu*	Wenchang, Hainan
TC-1	*Pantoea ananatis*	PV759632	Areca palm *A*. *catechu*	Tunchang, Hainan

**Table 4:** Reference sequences used in this study.

Reference Strain	GenBank Accession Number	Application
‘*Ca.* P. asteris’ 16SrI	FJ998269	Primer Design/Phylogenetic analysis
‘*Ca.* P. asteris’ 16SrI	FJ694685	Primer Design/Phylogenetic analysis
‘*Ca.* P. australasaticum’ 16SrII	OQ586085	Primer Design
*Areca catechu* chloroplast	NC_050163	Primer Design
*Burkholderia andropogonis*	NR_104960	Primer Design
*Pantoea ananatis*	MW174802	Primer Design
*Chrysophyllum albidum*	LC110196	Primer Design
*Curtobacterium citreum*	MF319766	Primer Design
*Curtobacterium luteum*	JX437941	Primer Design
*Sphingomonas yantingensis*	MF101149	Primer Design
*Bacillus cereus* (*Robbsia andropogonis*)	HQ833025	Primer Design
*Staphylococcus epidermidis*	CP040883	Primer Design
*Xanthomonas sacchari*	MN889285	Primer Design
*Xanthomonas campestris*	JX415480	Primer Design
‘*Ca.* P. aurantifolia’ WBDL 16SrII	U15442	Primer Design
‘*Ca.* P. australasiae’ PpYC 16SrII	Y10097	Primer Design
‘*Ca.* P. pruni’ PX11CT1 rrnA 16SrIII	JQ044393	Primer Design
‘*Ca.* P. ziziphi’ JWB-G1 16SrV	AB052876	Primer Design
‘*Ca.* P. ulmi’ EY1 16SrV	AY197655	Primer Design
‘*Ca*. P. rubi’ RuS 16SrV	AY197648	Primer Design
‘*Ca.* P. trifolii’ CP 16SrVI	AY390261	Primer Design
‘*Ca.* P. sudamericanum’ PassWB-Br3 16SrVI	GU292081	Primer Design
‘*Ca.* P. fraxini’ AshY1 16SrVII	AF092209	Primer Design
‘*Ca.* P. luffae’ LfWB rrnA 16SrVIII	AF248956	Primer Design
‘*Ca.* P. luffae’ LfWB rrnB 16SrVIII	AF353090	Primer Design
‘*Ca.* P. phoenicium’ AlmWB 16SrIX	AF515636	Primer Design
‘*Ca.* P. pyri’ PD1 16SrX	AJ542543	Primer Design
‘*Ca.* P. spartii’ SpaWB 16SrX	X92869	Primer Design
‘*Ca.* P. mali’ AP15 16 SrX	AJ542541	Primer Design
‘*Ca.* P. oryzae’ RYD-Th 16SrXI	AB052873	Primer Design
‘*Ca.* P. cirsii’ CirYS 16SrXI	KR869146	Primer Design
‘*Ca.* P. solani’ STOL11 16SrXII	AF248959	Primer Design
‘*Ca.* P. japonicum’ JPH 16SrXII	AB010425	Primer Design
‘*Ca.* P. convolvuli’ BY-S5711 16SrXII	JN833705	Primer Design
‘*Ca.* P. hispanicum’ MPV 16SrXIII	AF248960	Primer Design
‘*Ca.* P. meliae’ ChTY-Mo3 16SrXIII	KU850940	Primer Design
‘*Ca.* P. cynodontis’ BGWL-C1 16SrXIV	AJ550984	Primer Design
‘*Ca.* P. brasiliense’ HibWB26 16SrXV	AF147708	Primer Design
‘*Ca.* P. graminis’ SCYLP 16SrXVI	AY725228	Primer Design
‘*Ca.* P. caricae’ PAY 16SrXVII	AY725234	Primer Design
‘*Ca.* P. castaneae’ CnWB 16SrXIX	AB054986	Primer Design
‘*Ca.* P. omanense’ IM-1 16SrXXIX	EF666051	Primer Design
‘*Ca.* P. costaricanum’ SoyST1c1 16SrXXXI	HQ225630	Primer Design
‘*Ca.* P. malaysianum’ MaPV 16SrXXXII	OP237525	Primer Design
‘*Ca.* P. malaysianum’ MaPV 16SrXXXII	EU371934	Primer Design
‘*Ca.* P. wodyetiae’ FPYD Bangi-2 16SrXXXVI	KC844879	Primer Design
*Acholeplasma laidlawii*	CP000896	Phylogenetic analysis

## Data Availability

DNA sequences are available in the GenBank database, with the accession numbers listed in the Results. All other relevant data are within the paper and Appendix A.

## Abbreviations

The following abbreviations are used in this manuscript:

YLD	yellow leaf disease of areca palm
APYL	areca palm yellow leaf phytoplasma
LAMP	loop-mediated isothermal amplification
qPCR	quantitative PCR
ddPCR	droplet digital PCR

## Appendix A

**Table A1:** Areca palm leaf samples used in the detection assays using universal nested PCR in this study.

No.	Sample	Result	City/County	Year	No.	Sample	Result	City/County	Year
1	J069	Positive	Wenchang	2022	169	ZB1-2	Negative	Wenchang	2022
2	A056	Positive	Wenchang	2022	170	MQR4	Negative	Ledong	2021
3	T40	Positive	Wenchang	2022	171	HL3-4	Negative	Ledong	2021
4	W4	Positive	Wenchang	2022	172	HL3-1	Negative	Ledong	2021
5	S97	Positive	Wenchang	2022	173	HL1-2	Negative	Ledong	2021
6	M25	Positive	Wenchang	2023	174	HZ3-2	Negative	Ledong	2021
7	H61	Positive	Wenchang	2023	175	HZ5-2	Negative	Ledong	2021
8	H64	Positive	Wenchang	2023	176	T52	Negative	Ledong	2021
9	R71	Positive	Waning	2022	177	NY1-2	Negative	Wenchang	2022
10	R72	Positive	Waning	2022	178	ZB2-2	Negative	Wenchang	2022
11	R61	Negative	Waning	2022	179	M22	Negative	Wenchang	2022
12	R62	Negative	Waning	2022	180	M23	Negative	Wenchang	2022
13	R63	Negative	Waning	2022	181	M25	Negative	Wenchang	2022
14	R64	Negative	Waning	2022	182	M26	Negative	Wenchang	2022
15	R65	Negative	Waning	2022	183	M27	Negative	Wenchang	2022
16	R66	Negative	Waning	2022	184	M30	Negative	Wenchang	2022
17	R67	Negative	Waning	2022	185	M31	Negative	Wenchang	2022
18	R68	Negative	Waning	2022	186	L30	Negative	Wenchang	2021
19	R69	Negative	Waning	2022	187	L32	Negative	Wenchang	2021
20	R70	Negative	Waning	2022	188	L33	Negative	Wenchang	2021
21	R73	Negative	Waning	2022	189	S40	Negative	Wenchang	2023
22	R74	Negative	Waning	2022	190	S41	Negative	Wenchang	2023
23	R75	Negative	Waning	2022	191	S42	Negative	Wenchang	2023
24	R76	Negative	Waning	2022	192	S43	Negative	Wenchang	2023
25	R77	Negative	Waning	2022	193	S44	Negative	Wenchang	2023
26	R78	Negative	Waning	2022	194	S45	Negative	Wenchang	2023
27	R79	Negative	Waning	2022	195	S46	Negative	Wenchang	2023
28	R80	Negative	Waning	2022	196	S47	Negative	Wenchang	2023
29	R81	Negative	Waning	2022	197	S50	Negative	Wenchang	2023
30	R82	Negative	Waning	2022	198	W11	Negative	Wenchang	2023
31	R83	Negative	Waning	2022	199	W12	Negative	Wenchang	2023
32	R84	Negative	Waning	2022	200	W13	Negative	Wenchang	2023
33	R85	Negative	Waning	2022	201	W14	Negative	Wenchang	2023
34	R86	Negative	Waning	2022	202	W15	Negative	Wenchang	2023
35	R87	Negative	Waning	2022	203	W16	Negative	Wenchang	2023
36	R88	Negative	Waning	2022	204	W17	Negative	Wenchang	2023
37	R89	Negative	Waning	2022	205	W18	Negative	Wenchang	2023
38	R90	Negative	Waning	2022	206	W19	Negative	Wenchang	2023
39	W1	Negative	Waning	2022	207	W20	Negative	Wenchang	2023
40	W2	Negative	Wenchang	2022	208	W21	Negative	Wenchang	2023
41	W3	Negative	Wenchang	2022	209	W22	Negative	Wenchang	2023
42	W3-4	Negative	Wenchang	2022	210	W23	Negative	Wenchang	2023
43	A09	Negative	Wenchang	2022	211	W24	Negative	Wenchang	2023
44	A12	Negative	Wenchang	2022	212	W25	Negative	Wenchang	2023
45	A13	Negative	Wenchang	2022	213	W26	Negative	Wenchang	2023
46	A14	Negative	Wenchang	2022	214	W27	Negative	Wenchang	2023
47	A16	Negative	Wenchang	2022	215	W28	Negative	Wenchang	2023
48	A19	Negative	Wenchang	2022	216	W29	Negative	Wenchang	2023
49	A22	Negative	Wenchang	2022	217	W30	Negative	Wenchang	2023
50	A24	Negative	Wenchang	2022	218	W31	Negative	Wenchang	2023
51	A27	Negative	Wenchang	2022	219	W32	Negative	Wenchang	2023
52	A30	Negative	Wenchang	2022	220	W33	Negative	Wenchang	2023
53	A31	Negative	Wenchang	2022	221	W34	Negative	Wenchang	2023
54	A32	Negative	Wenchang	2022	222	W35	Negative	Wenchang	2023
55	A34	Negative	Wenchang	2022	223	W36	Negative	Wenchang	2023
56	A36	Negative	Wenchang	2022	224	W37	Negative	Wenchang	2023
57	A37	Negative	Wenchang	2022	225	W38	Negative	Wenchang	2023
58	A39	Negative	Wenchang	2022	226	W39	Negative	Wenchang	2023
59	A49	Negative	Wenchang	2022	227	B2-1	Negative	Baoting	2021
60	A50	Negative	Wenchang	2022	228	B2-2	Negative	Baoting	2021
61	A51	Negative	Wenchang	2022	229	B2-4	Negative	Baoting	2021
62	A55	Negative	Wenchang	2022	230	B2-5	Negative	Baoting	2021
63	A57	Negative	Wenchang	2022	231	B2-6	Negative	Baoting	2021
64	A59	Negative	Wenchang	2022	232	B2-7	Negative	Baoting	2021
65	A60	Negative	Wenchang	2022	233	B2-9	Negative	Baoting	2021
66	B13	Negative	Baoting	2020	234	B4-1	Negative	Baoting	2021
67	B4	Negative	Baoting	2020	235	B4-2	Negative	Baoting	2021
68	B17	Negative	Baoting	2020	236	B4-3	Negative	Baoting	2021
69	B20	Negative	Baoting	2020	237	B4-4	Negative	Baoting	2021
70	B21	Negative	Baoting	2020	238	B4-5	Negative	Baoting	2021
71	B23	Negative	Baoting	2020	239	B4-6	Negative	Baoting	2021
72	B24	Negative	Baoting	2020	240	B4-7	Negative	Baoting	2021
73	B25	Negative	Baoting	2020	241	B4-8	Negative	Baoting	2021
74	B27	Negative	Baoting	2020	242	B4-9	Negative	Baoting	2021
75	B30	Negative	Baoting	2020	243	B4-10	Negative	Baoting	2021
76	B31	Negative	Baoting	2020	244	B4-11	Negative	Baoting	2021
77	B32	Negative	Baoting	2020	245	B4-12	Negative	Baoting	2021
78	B33	Negative	Baoting	2020	246	B4-13	Negative	Baoting	2021
79	B37	Negative	Baoting	2020	247	B4-14	Negative	Baoting	2021
80	B41	Negative	Baoting	2020	248	B4-15	Negative	Baoting	2021
81	B44	Negative	Baoting	2020	249	B4-16	Negative	Baoting	2021
82	B46	Negative	Baoting	2020	250	B4-17	Negative	Baoting	2021
83	B47	Negative	Baoting	2020	251	B4-18	Negative	Baoting	2021
84	B49	Negative	Baoting	2020	252	B4-19	Negative	Baoting	2021
85	B53	Negative	Baoting	2020	253	B5-4	Negative	Baoting	2021
86	B54	Negative	Baoting	2020	254	B5-5	Negative	Baoting	2021
87	B56	Negative	Baoting	2020	255	B5-6	Negative	Baoting	2021
88	B57	Negative	Baoting	2020	256	B5-10	Negative	Baoting	2021
89	B61	Negative	Baoting	2020	257	B6-1	Negative	Baoting	2021
90	B63	Negative	Baoting	2020	258	B6-2	Negative	Baoting	2021
91	B76	Negative	Baoting	2020	259	B6-4	Negative	Baoting	2021
92	C21	Negative	Qionghai	2020	260	B6-5	Negative	Baoting	2021
93	C26	Negative	Qionghai	2020	261	B6-6	Negative	Baoting	2021
94	C27	Negative	Qionghai	2020	262	B6-8	Negative	Baoting	2021
95	C41	Negative	Qionghai	2020	263	B6-10	Negative	Baoting	2021
96	C47	Negative	Qionghai	2020	264	B6-11	Negative	Baoting	2021
97	C61	Negative	Qionghai	2020	265	F04-2	Negative	Tunchang	2020
98	C68	Negative	Qionghai	2020	266	F05-2	Negative	Tunchang	2020
99	C69	Negative	Qionghai	2020	267	F05-1	Negative	Tunchang	2020
100	C70	Negative	Qionghai	2020	268	F06-1	Negative	Tunchang	2020
101	C73	Negative	Qionghai	2020	269	F07-1	Negative	Tunchang	2020
102	C85	Negative	Qionghai	2020	270	F022-1	Negative	Tunchang	2020
103	C87	Negative	Qionghai	2020	271	F028-2	Negative	Tunchang	2020
104	C89	Negative	Qionghai	2020	272	F023-2	Negative	Tunchang	2020
105	C90	Negative	Qionghai	2020	273	F035-1	Negative	Tunchang	2020
106	C91	Negative	Qionghai	2020	274	F036-1	Negative	Tunchang	2020
107	C95	Negative	Qionghai	2020	275	F036-2	Negative	Tunchang	2020
108	C99	Negative	Qionghai	2020	276	F037-1	Negative	Tunchang	2020
109	C100	Negative	Qionghai	2020	277	F037-2	Negative	Tunchang	2020
110	BSL-1	Negative	Ding’an	2023	278	F038-1	Negative	Tunchang	2020
111	BSL-2	Negative	Ding’an	2023	279	F038-2	Negative	Tunchang	2020
112	BSL-3	Negative	Ding’an	2023	280	F039-1	Negative	Tunchang	2020
113	BSL-4	Negative	Ding’an	2023	281	F040-1	Negative	Tunchang	2020
114	BSL-5	Negative	Ding’an	2023	282	F040-2	Negative	Tunchang	2020
115	BSL-6	Negative	Ding’an	2023	283	F041-2	Negative	Tunchang	2020
116	BSL-7	Negative	Ding’an	2023	284	F044-1	Negative	Tunchang	2020
117	BSL-8	Negative	Ding’an	2023	285	F044-2	Negative	Tunchang	2020
118	BSL-9	Negative	Ding’an	2023	286	F045-1	Negative	Tunchang	2020
119	BSL-10	Negative	Ding’an	2023	287	F045-2	Negative	Tunchang	2020
120	BSL-11	Negative	Ding’an	2023	288	F049-2	Negative	Tunchang	2020
121	BSL-12	Negative	Ding’an	2023	289	F055-2	Negative	Tunchang	2020
122	BSL-13	Negative	Ding’an	2023	290	F099-1	Negative	Tunchang	2020
123	BSL-14	Negative	Ding’an	2023	291	C002-1	Negative	Qionghai	2020
124	BSL-15	Negative	Ding’an	2023	292	C007-2	Negative	Qionghai	2020
125	BSL-16	Negative	Ding’an	2023	293	C013-2	Negative	Qionghai	2020
126	BSL-17	Negative	Ding’an	2023	294	C018Y-1	Negative	Qionghai	2020
127	BSL-18	Negative	Ding’an	2023	295	C018Y-2	Negative	Qionghai	2020
128	BSL-19	Negative	Ding’an	2023	296	C021-1	Negative	Qionghai	2020
129	BSL-20	Negative	Ding’an	2023	297	C023-1	Negative	Qionghai	2020
130	XS-1	Negative	Ding’an	2023	298	C026-1	Negative	Qionghai	2020
131	XS-2	Negative	Ding’an	2023	299	C026-2	Negative	Qionghai	2020
132	XS-3	Negative	Ding’an	2023	300	C031-2	Negative	Qionghai	2020
133	XS-4	Negative	Ding’an	2023	301	C048-1	Negative	Qionghai	2020
134	XS-5	Negative	Ding’an	2023	302	C064-1	Negative	Qionghai	2020
135	XS-6	Negative	Ding’an	2023	303	C065-2	Negative	Qionghai	2020
136	XS-7	Negative	Ding’an	2023	304	C071-2	Negative	Qionghai	2020
137	XS-8	Negative	Ding’an	2023	305	C072-2	Negative	Qionghai	2020
138	XS-9	Negative	Ding’an	2023	306	C076-2	Negative	Qionghai	2020
139	XS-10	Negative	Ding’an	2023	307	C077-2	Negative	Qionghai	2020
140	XS-11	Negative	Ding’an	2023	308	C078-1	Negative	Qionghai	2020
141	LK-1	Negative	Ding’an	2023	309	C080-2	Negative	Qionghai	2020
142	LK-2	Negative	Ding’an	2023	310	C056-2	Negative	Qionghai	2020
143	LK-3	Negative	Ding’an	2023	311	C065-2	Negative	Qionghai	2020
144	LK-4	Negative	Ding’an	2023	312	D008-2	Negative	Ding’an	2020
145	LK-5	Negative	Ding’an	2023	313	D039-1	Negative	Ding’an	2020
146	LK-6	Negative	Ding’an	2023	314	D062-2	Negative	Ding’an	2020
147	LK-7	Negative	Ding’an	2023	315	D093-2	Negative	Ding’an	2020
148	LK-8	Negative	Ding’an	2023	316	ZY2-1	Negative	Wenchang	2020
149	LK-9	Negative	Ding’an	2023	317	7-4-2	Negative	Wenchang	2020
150	LK-10	Negative	Ding’an	2023	318	003-2	Negative	Wenchang	2020
151	LK-11	Negative	Ding’an	2023	319	B021-2	Negative	Baoting	2020
152	LK-12	Negative	Ding’an	2023	320	B027-2	Negative	Baoting	2020
153	LK-13	Negative	Ding’an	2023	321	B029-2	Negative	Baoting	2020
154	LK-14	Negative	Ding’an	2023	322	B030-1	Negative	Baoting	2020
155	LK-15	Negative	Ding’an	2023	323	B032-1	Negative	Baoting	2020
156	LK-16	Negative	Ding’an	2023	324	B037-2	Negative	Baoting	2020
157	LK-17	Negative	Ding’an	2023	325	B038-2	Negative	Baoting	2020
158	LK-18	Negative	Ding’an	2023	326	B042-1	Negative	Baoting	2020
159	LK-19	Negative	Ding’an	2023	327	B042-2	Negative	Baoting	2020
160	LK-20	Negative	Ding’an	2023	328	B055-2	Negative	Baoting	2020
161	I027	Negative	Qionghai	2022	329	B059-1	Negative	Baoting	2020
162	N01	Negative	Lingshui	2022	330	B059-2	Negative	Baoting	2020
163	N06	Negative	Lingshui	2022	331	B056-1	Negative	Baoting	2020
164	N07	Negative	Lingshui	2022	332	B056-2	Negative	Baoting	2020
165	N012	Negative	Lingshui	2022	333	B061-1	Negative	Baoting	2020
166	N013	Negative	Lingshui	2022	334	B069-1	Negative	Baoting	2020
167	ZH-40	Negative	Lingshui	2022	335	B069-2	Negative	Baoting	2020
168	NLT	Negative	Lingshui	2022					

Note: a total of 10 leaf samples were positive in the detection using the developed nested PCR.
